# Enhanced Production, Purification, Characterization and Mechanism of Action of Salivaricin 9 Lantibiotic Produced by *Streptococcus salivarius* NU10

**DOI:** 10.1371/journal.pone.0077751

**Published:** 2013-10-16

**Authors:** Abdelahhad Barbour, Koshy Philip, Sekaran Muniandy

**Affiliations:** 1 Institute of Biological Sciences, Microbiology Division, Faculty of Science, University of Malaya, Kuala Lumpur, Malaysia; 2 Department of Molecular Medicine, Faculty of Medicine, University of Malaya, Kuala Lumpur, Malaysia; Centre National de la Recherche Scientifique, Aix-Marseille Université, France

## Abstract

**Background:**

Lantibiotics are small lanthionine-containing bacteriocins produced by lactic acid bacteria. Salivaricin 9 is a newly discovered lantibiotic produced by *Streptococcus salivarius*. In this study we present the mechanism of action of salivaricin 9 and some of its properties. Also we developed new methods to produce and purify the lantibiotic from strain NU10.

**Methodology / Principal Findings:**

Salivaricin 9 was found to be auto-regulated when an induction assay was applied and this finding was used to develop a successful salivaricin 9 production system in liquid medium. A combination of XAD-16 and cation exchange chromatography was used to purify the secondary metabolite which was shown to have a molecular weight of approximately 3000 Da by SDS-PAGE. MALDI-TOF MS analysis indicated the presence of salivaricin 9, a 2560 Da lantibiotic. Salivaricin 9 is a bactericidal molecule targeting the cytoplasmic membrane of sensitive cells. The membrane permeabilization assay showed that salivaricin 9 penetrated the cytoplasmic membrane and induced pore formation which resulted in cell death. The morphological changes of test bacterial strains incubated with salivaricin 9 were visualized using Scanning Electron Microscopy which confirmed a pore forming mechanism of inhibition. Salivaricin 9 retained biological stability when exposed to high temperature (90-100°C) and stayed bioactive at pH ranging 2 to 10. When treated with proteinase K or peptidase, salivaricin 9 lost all antimicrobial activity, while it remained active when treated with lyticase, catalase and certain detergents.

**Conclusion:**

The mechanism of antimicrobial action of a newly discovered lantibiotic salivaricin 9 was elucidated in this study. Salivaricin 9 penetrated the cytoplasmic membrane of its targeted cells and induced pore formation. This project has given new insights on lantibiotic peptides produced by *S. salivarius* isolated from the oral cavities of Malaysian subjects.

## Introduction

Different genera of lactic acid bacteria (LAB) can produce different kinds of antimicrobial peptides and bacteriocins such as plantaricin produced by *Lactobacillus plantarum* [1], enterococcin produced by *Enterococcus faecium* [2], leucocin produced by *Leuconostoc carnosum* [3], pediocin produced by *Pediococcus acidilactici* [4] and others. Interest in bacteriocins has increased recently due to their antimicrobial activity towards Gram-positive pathogens [5-7]. Bacteriocins produced by oral cavity microorganisms have been reported previously [8-12]. Most of the bacteriocins produced by human oral streptococci are controlled by quorum sensing whereby bacteria can only produce the bioactive bacteriocin when grown on solid or semi-solid medium supplemented with agar or agarose [8,9,13]. Lantibiotics are heat-stable, post-translationally modified, ribosomally synthesized small antimicrobial peptides which contain lanthionine (Lan) and/or β-methyllanthionine (MeLan) residues [14-16]. Some lantibiotics (e.g. nisin) are produced in significant quantity when the producer is growing in liquid medium [17-19]. In recent years methods to enhance and optimize bacteriocin production have been developed [20-22] due to the potential importance of bacteriocin-producing strains in replacement therapy [23]. Many bacteriocin-producing strains have already been used as probiotics. *S. salivarius* K12 is an oral probiotic producing two kinds of antimicrobial peptides referred as salivaricin A2 and salivaricin B [9]. Both bacteriocins can be recovered by a freeze-thaw extraction method after the producer is grown on solid medium. Pore formation is a common mode of action of lantibiotics [24,25]. The permeabilization of the cytoplasmic membrane of targeted cells has been studied to investigate whether bioactive lantibiotics can penetrate the cell membrane of certain potential pathogens [26,27]. In this study we developed a new induction assay to produce salivaricin 9 in liquid medium for the first time using *S. salivarius* strain NU10 isolated from a Malaysian subject. The purification method used to recover salivaricin 9 was XAD-16 chromatography followed by cation exchange chromatography. Tris-Tricine SDS PAGE indicated that the peptide has a molecular weight of approximately 3,000 Da. Matrix assisted laser desorption ionization time of flight mass spectrometry MALDI-TOF (MS) analysis indicated a molecular weight of 2560 Da. We also studied the mechanism of action of the pure salivaricin 9 using SYTOX® Green. Flow cytometry analysis was also used to demonstrate membrane disruption using propidium iodide to probe cells with compromised membranes. Scanning electron microscopy was used to detect the morphological changes of the targeted indicator microorganisms after treating with salivaricin 9. Investigating the mechanism of action of lantibiotics produced by oral streptococci can assist with the development of new antimicrobials and probiotics that can be used to enhance the health of the human oral cavity and upper respiratory tract. 

## Results

### Simultaneous antagonism test

**Figure 1:**
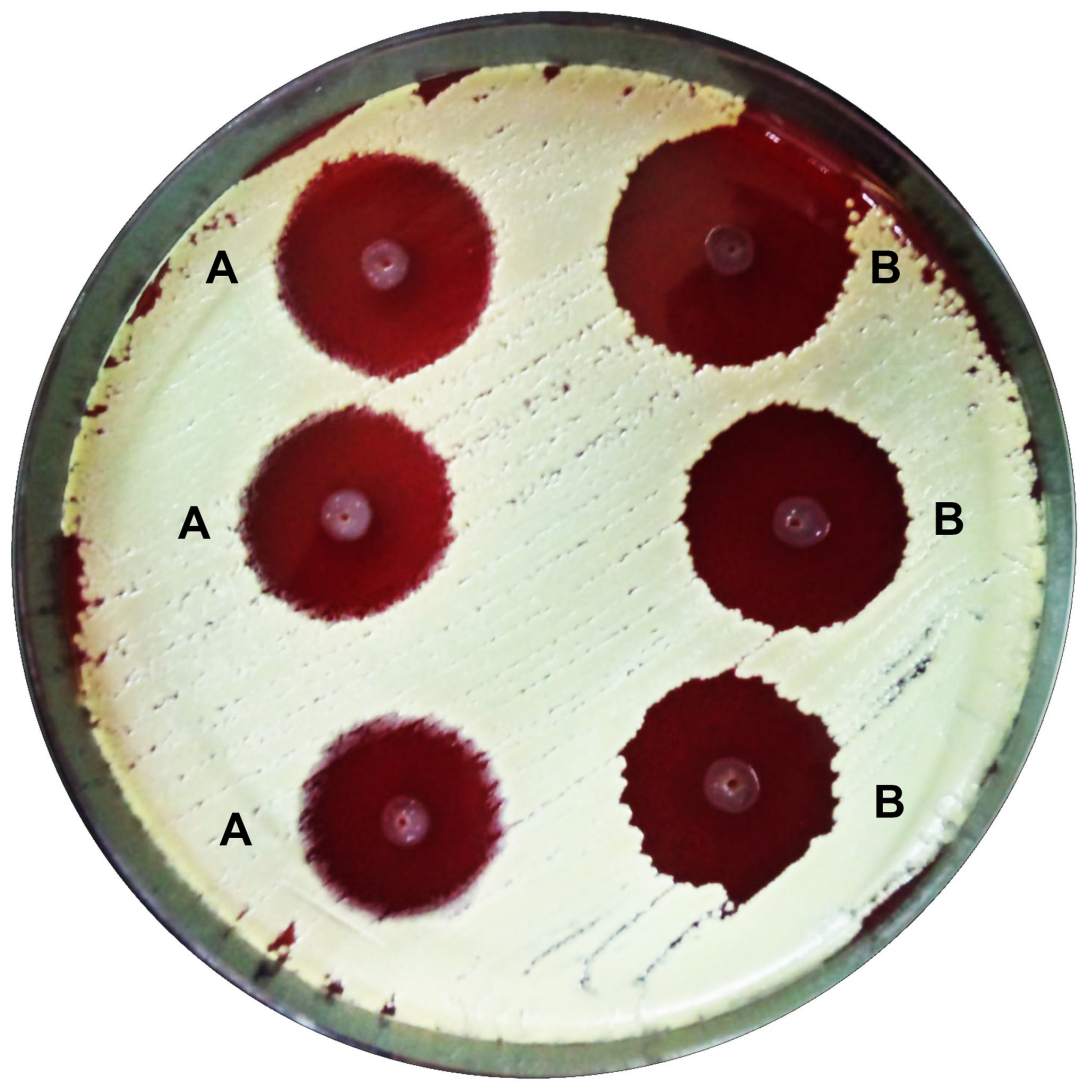
Simultaneous Antagonism Assay. A: NU10 was used as a bacteriocin producer, B: K12 (producer of salivaricin A and salivaricin B) was used as a positive control. *Micrococcus luteus* was used as target indicator strain.

Strain NU10 isolated from a Malaysian subject showed significant inhibitory activity when tested in the simultaneous antagonism test. When both producer and indicator were grown at the same time on blood agar, the producer strain NU10 inhibited the indicator growth. Figure 1 shows a comparison of the inhibition zones caused by strains NU10 and K12 (a commercial probiotic). 

### Distribution of *salA* and *sivA* structural genes in strains NU10 and YU10

**Figure 2:**
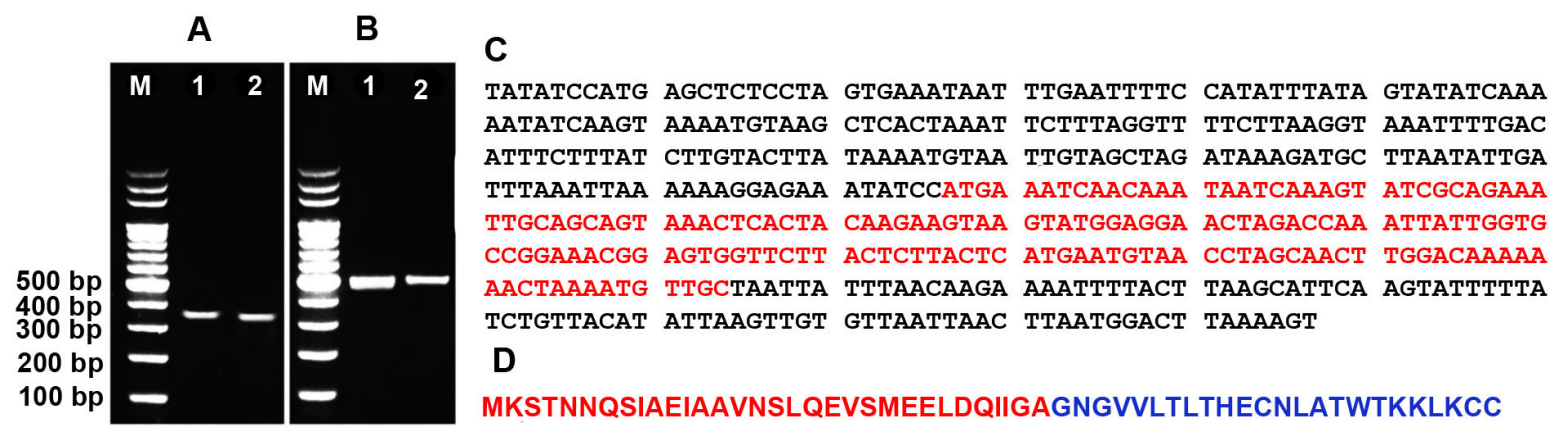
Gene encoding salivaricin 9 production. A: *salA* structural gene encoding salivaricin A production in strains NU10 (1) and YU10 (2). B: *sivA* structural gene encoding salivaricin 9 production in strains NU10 (1) and YU10 (2). (M) 100 bp DNA leader. Gel electrophoresis was performed using 2% (w/v) agarose and stained using GelRed^TM^. C: Assembled *sivA* gene sequence. The open reading frame ORF encoding the production of the leader and mature peptide is highlighted in red. D: *In* *silico* DNA to protein translation, leader peptide (red) and mature salivaricin 9 (blue).

Both strains NU10 and YU10 were shown to harbour the structural genes *sivA* and *salA* encoding the production of salivaricin 9 and A respectively. *sivA* from strain NU10 was sequenced and translated to protein using *in silico* analysis [28] (Figure 2). The *sivA* sequence of strain NU10 showed 100% homology with *sivA* of strain 9 (accession number: DQ889747.1).

### Auto-inducing and cross inducing activities of salivaricins 9 and A

Bacteriocins produced by *S. salivarius* are often not expressed in liquid media. In this study, we tried to enhance bacteriocin production by using specific induction. Table 1 shows that salivaricin 9 production appeared to be auto-regulated. When salivaricin 9 was added to NU10 cultures it induced the production of antimicrobial activity. This auto-induction capability was used to enhance salivaricin 9 production in liquid medium. Crude bacteriocin preparations from all salivaricin producers were designated as BLIS (BLIS-NU10, BLIS-YU10 and BLIS-K12) and each of these preparations contains more than one kind of bacteriocin molecule that was tested as an inducer in this study. BLIS-NU10 extracted from strain NU10 cells contained salivaricins A and 9 and it induced bacteriocin production in strains YU10 and K12 since all *S. salivarius* strains tested in this study harbour *salA* structural gene encoding the production of salivaricin A. The pure FPLC-fraction of salivaricin 9 was also used as an inducer and was shown to induce bacteriocin production only in strains NU10 and YU10 as both harbour the *sivA* gene encoding salivaricin 9 production. However, pure salivaricin 9 had no induction activity when incubated with strain K12, which is PCR-negative for the structural gene of salivaricin 9. Nisin did not show any induction activity when incubated with any of the *S. salivarius* strains but it induced the production of the inhibitory activity when incubated with nisin producer *Lactococcus lactis* strain ATCC11454 (Table 1).

**Table 1:** Induction of inhibitor production by *S. salivarius* strains NU10, YU10, K12 and nisin-producing strain ATCC11454 using crude preparations, purified salivaricin 9 and nisin.

**Inhibitor-positive preparation tested for inducing activity**	**Lantibiotic peptide(s) in preparation**	**Preparation induces inhibitor production in *S. salivarius* strains and nisin-producing strain**			
		**NU10**	**YU10**	**K12**	**ATCC11454** ^†^
BLIS-NU10 ^α^	Sal A & 9	Yes	Yes	Yes	No
Pure salivaricin 9 ^β^	Sal 9	Yes	Yes	No	No
BLIS-YU10 ^α^	Sal A & 9	Yes	Yes	Yes	No
BLIS-K12 ^α^	Sal A & B	Yes	Yes	Yes	No
Nisin (Sigma)	Nisin	No	No	No	Yes

α BLIS (bacteriocin-like inhibitory substances) representing the crude extract of each producer strain.

β FPLC-purified fraction of salivaricin 9 (sal9) produced by strain NU10.

† *Lactococcus lactis* strain (producer of nisin lantibiotic).

### Production of salivaricin 9 in liquid medium

**Figure 3:**
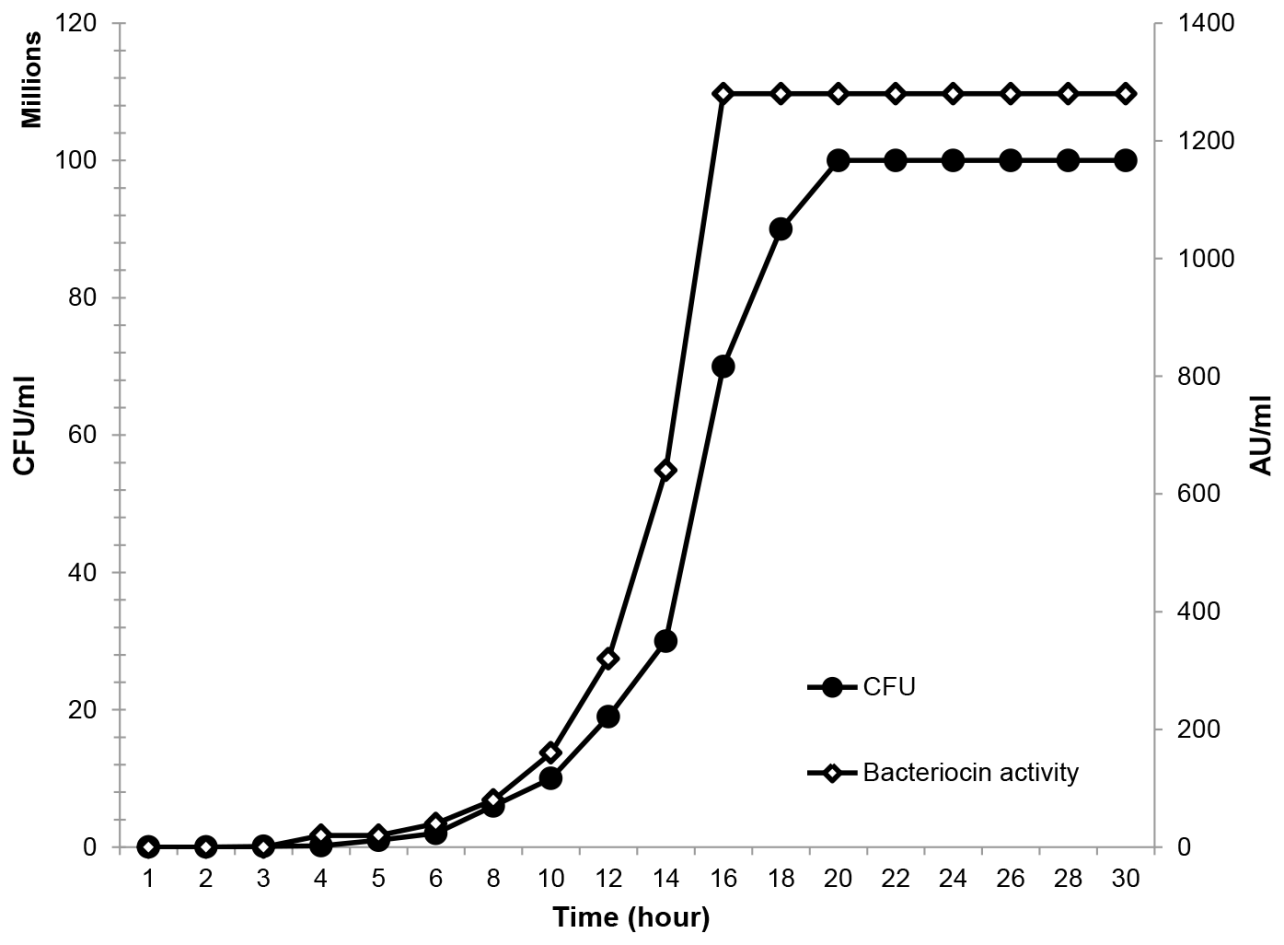
Growth kinetics of strain NU10 during salivaricin 9 production. Inhibitory activity of the cell free supernatant tested against *Micrococcus luteus*. Salivaricin 9 production was stable and consistent when strain NU10 reached the stationary phase of growth.

Induced cultures of *S. salivarius* NU10 showed detectable inhibitory activity. After 8 hours the level of salivaricin 9 started to increase gradually until it reached more than 1200 arbitrary units per millilitre (AU/ml) at 16 hours. Once the growth kinetics of strain NU10 reached the stationary phase, the levels of the inhibitory activity remained stable (Figure 3). The arbitrary units representing bacteriocin titre are expressed as the reciprocal of the highest dilution that showed inhibitory activity against indicator strain, *Micrococcus luteus*.

### Salivaricin 9 purification and minimum inhibitory concentration (MIC)

**Figure 4:**
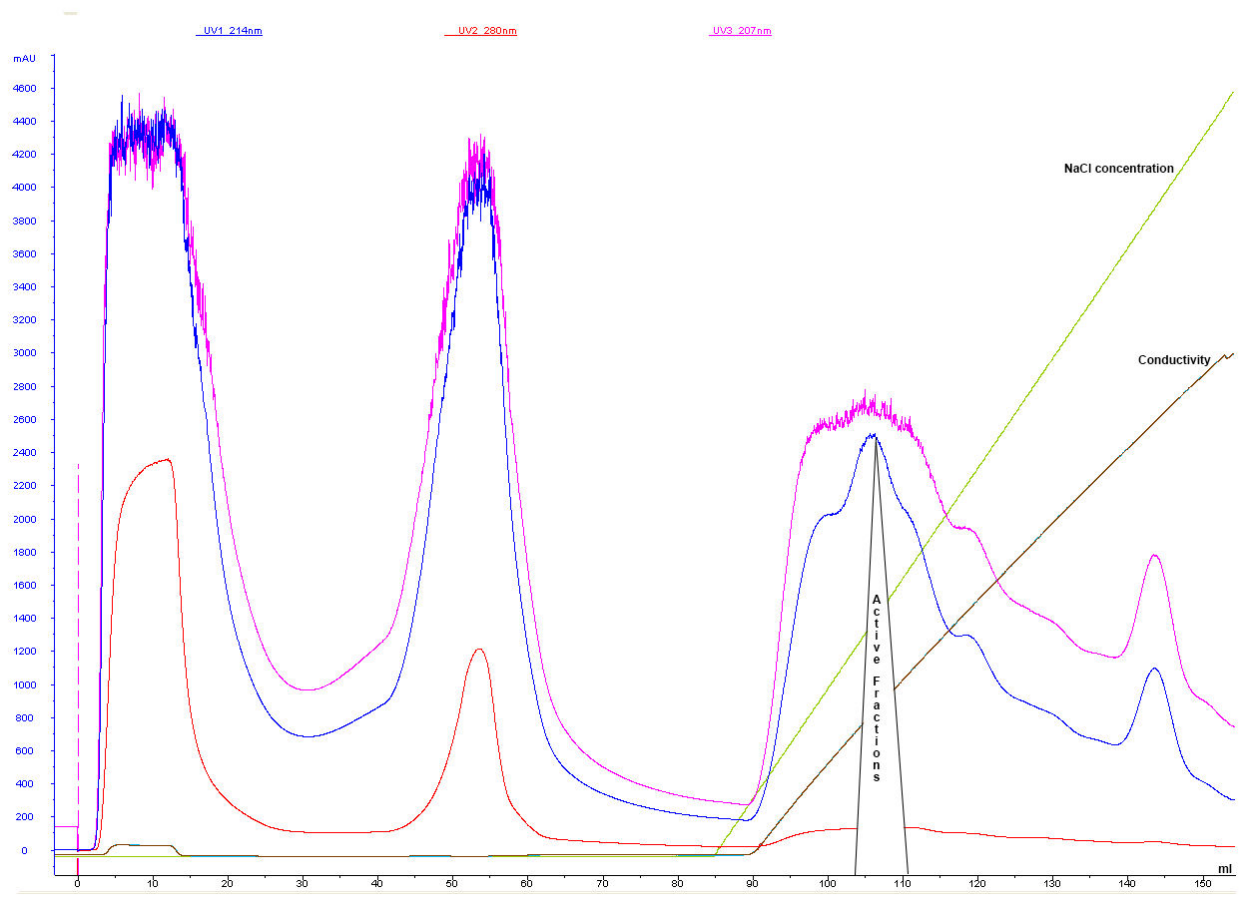
FPLC profile showing purification of salivaricin 9 using SP FF column. Salivaricin 9 was bound to the strong cation exchanger efficiently and eluted using linear gradient of increasing NaCl concentrations. Salivaricin 9 was detected only at wave lengths of 207 and 214 nm.

**Figure 5:**
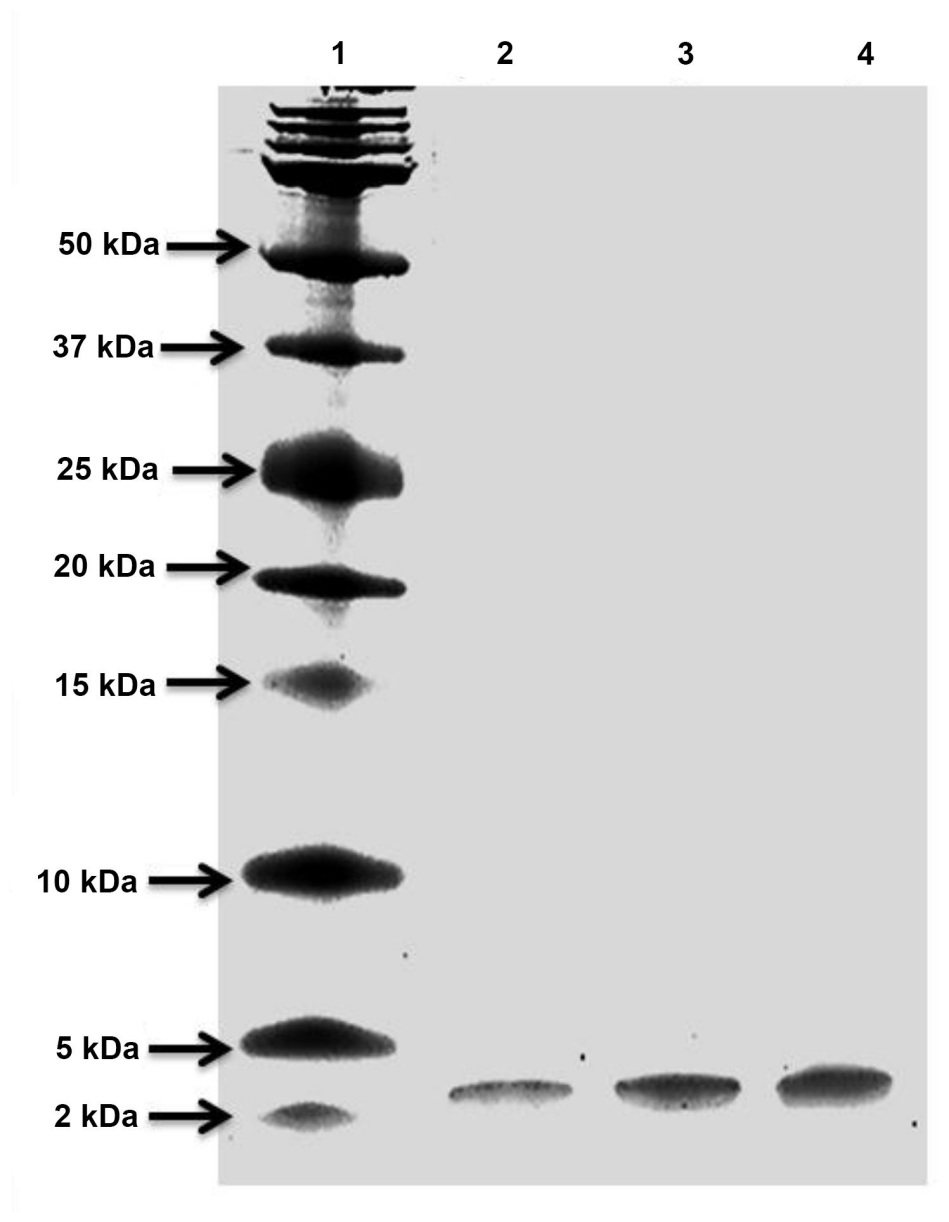
Tris-Tricine SDS page of the purified peptide. Lane 1: Dual Xtra protein marker (Bio Rad). Lanes: 2, 3 and 4: active fractions eluted from FPLC system.

XAD-16 and cation exchange chromatography were used to purify salivaricin 9 from liquid cultures. The inhibitory activity bound to the strong cation exchanger SP FF column very efficiently. Salivaricin 9 could not be detected at a UV wavelength of 280 nm (Figure 4) indicating a relative absence of aromatic amino acid residues in the peptide. Therefore, two additional wave lengths of 207 nm and 214 nm were used. The inhibitory activity started to elute from the column at 23% NaCl. Purification steps are described in Table 2. The three 1ml active fractions were tested on Tris-Tricine SDS PAGE to estimate the molecular weight and check for the purity of the final product. Each of the three active fractions showed single protein bands with a molecular weight of approximately 3,000 Da compared to the protein standard (Figure 5). *Micrococcus luteus* and *Corynebacterium* *spp* showed to be the most sensitive indicators to the salivaricin 9 peptide and the MIC values of these strains were low (4-8 µg.mL^-1^). *Streptococcus pyogenes* strain ATCC12344 was inhibited by a higher concentration of salivaricin 9 (MIC 32 µg.mL^-1^). MIC values of the different test bacteria are listed in Table 3. 

**Table 2:** Purification of salivaricin 9 using XAD-16 and cation exchange chromatography.

Step	Volume (ml)	Activity (AU/ml)	Total Protein (mg)	Total activity (AU.10^3^)	Specific activity (AU/mg)	Yield (%)	Purification (fold)
**CFS ^¥^**	900	1280	10000	1152	115.2	100	1
**XAD-16**	30	2560	180	76.8	426.6	6.6	3.7
**SP FF ^§^**	3	2560	0.008	7.6	96 x 10^4^	0.659	8333

¥ Cell free supernatant from induced culture

§ Strong cation exchanger column

**Table 3:** Simultaneous Antagonism Test and Minimal Inhibitory Concentration (MIC).

**Indicator microorganism**	**Simultaneous antagonism test**	**MIC (µg/ml) of salivaricin 9**
*Micrococcus luteus* ATCC10240	++++	4
*Micrococcus luteus* GAB13	+++	16
*Haemophilus parainfluenza* TONEJ11	-	-
*Actinomyces naeslundii* TG2	-	-
*Streptococcus equisimilis* ATCC12388	++	64
*Staphylococcus aureus* RF122	-	-
*Corynebacterium* *spp* GH17	++++	8
*Lactococcus lactis* ATCC11454	-	128
*Streptococcus mutans* GEJ11	-	-
*Streptococcus pyogenes* ATCC12344	-	32
*Streptococcus pyogenes* ATCC12384	-	32
*Bacillus cereus* ATCC14579	-	-

++++ inhibition zone >12 mm, +++ inhibition zone = 10 mm, ++ inhibition zone < 5 mm, - No inhibition.

### Salivaricin 9 identification

**Figure 6:**
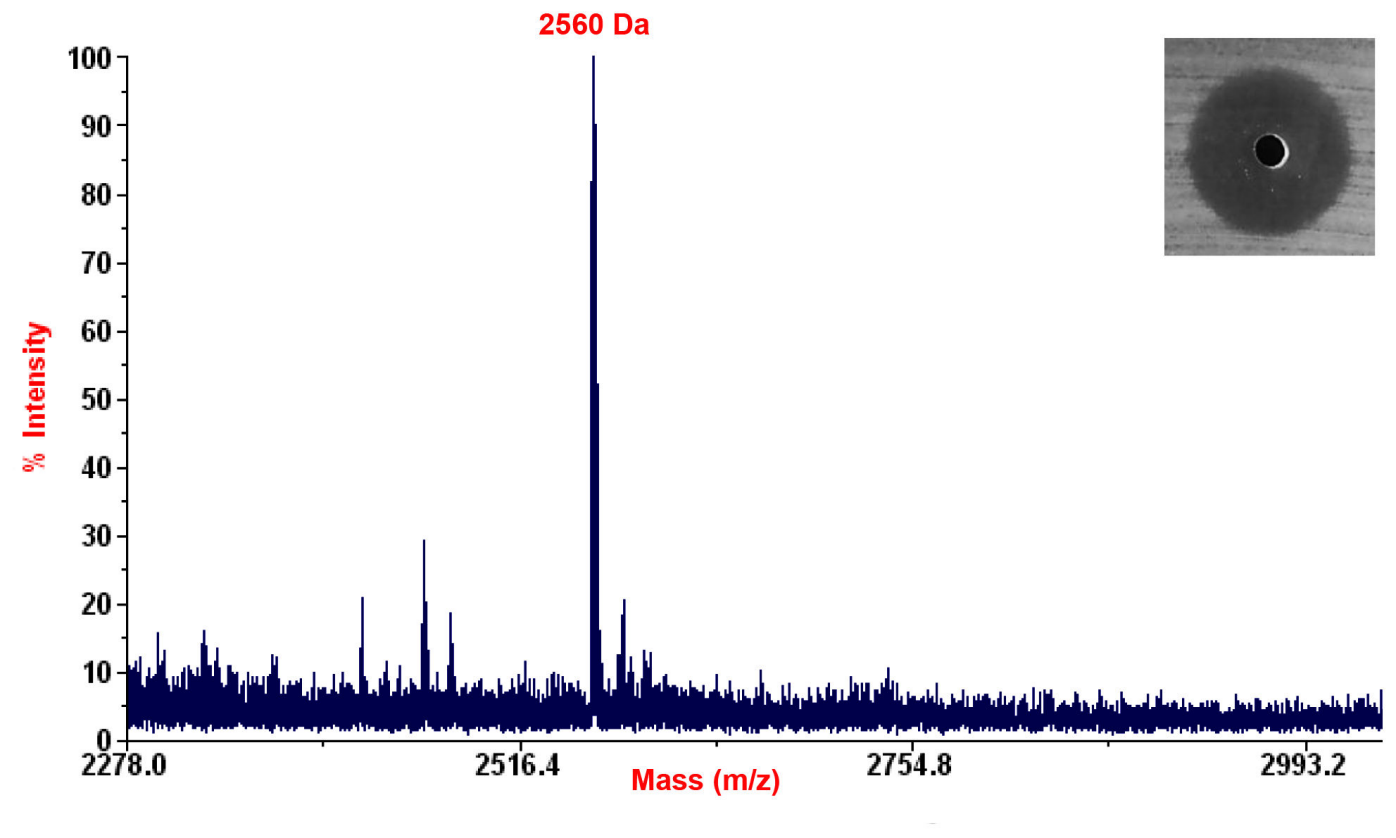
MALDI-TOF MS analysis of salivaricin 9. Active peak indicating the molecular weight of salivaricin 9 at 2560 Daltons.

Pure lantibiotic was subjected to MALDI-TOF (MS) analysis and showed the presence of a 2560 Da peptide representing salivaricin 9 (Figure 6).

### Salivaricin 9 mode of action

**Figure 7:**
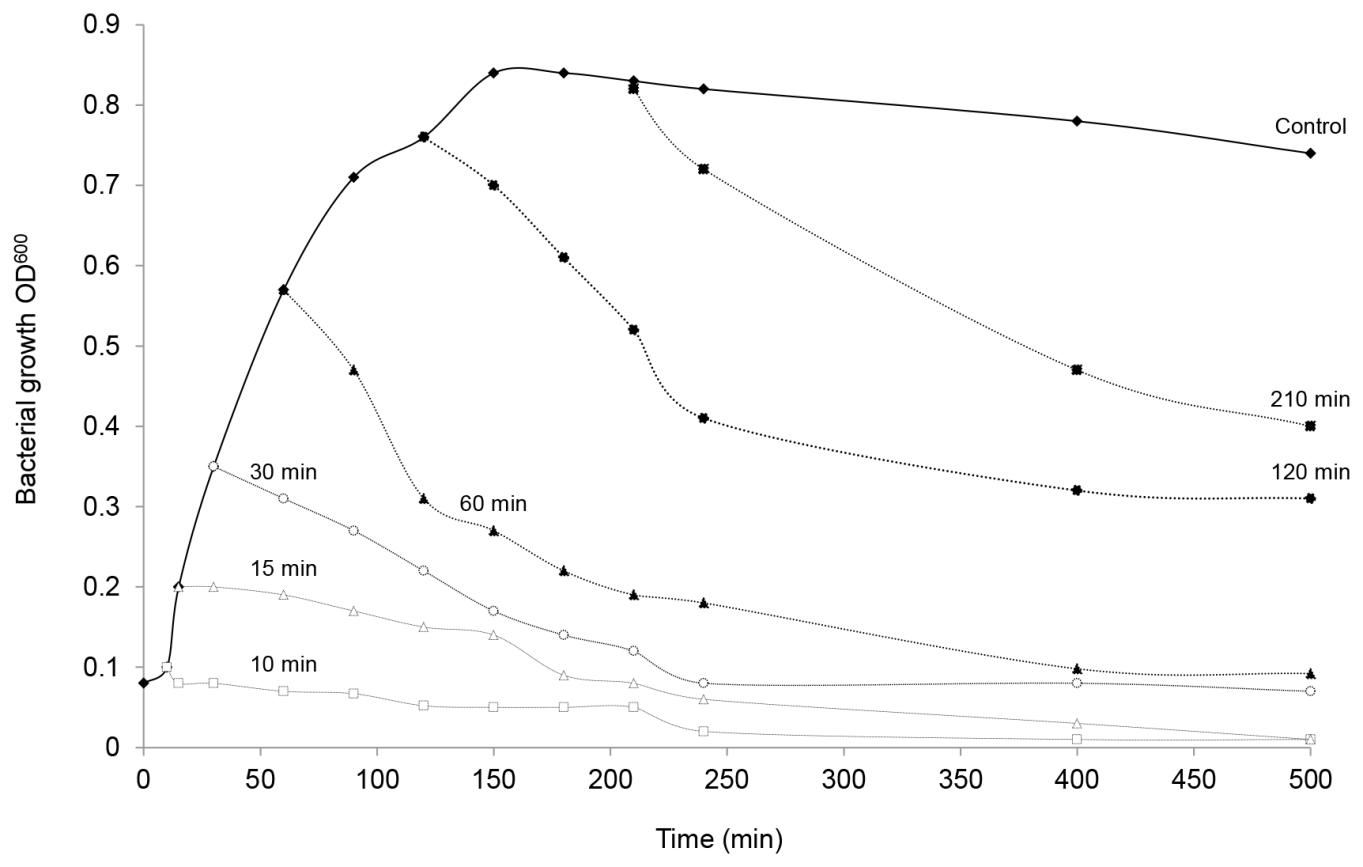
Bactericidal mode of action of salivaricin 9. Salivaricin 9 was added to different phases of bacterial growth. Salivaricin 9 induced bacterial lysis and decreased the indicator bacterial growth significantly. The sensitive bacteria *Micrococcus luteus* lost the ability to grow again after salivaricin 9 was added.

When added to different growth phases of *Micrococcus luteus* culture, salivaricin 9 induced cell lysis. Figure 7 shows the decreased OD_600_ reading when salivaricin 9 was added to the culture, indicating that salivaricin 9 is a bactericidal and bacteriolytic peptide. 

### Permeabilization assay

**Figure 8:**
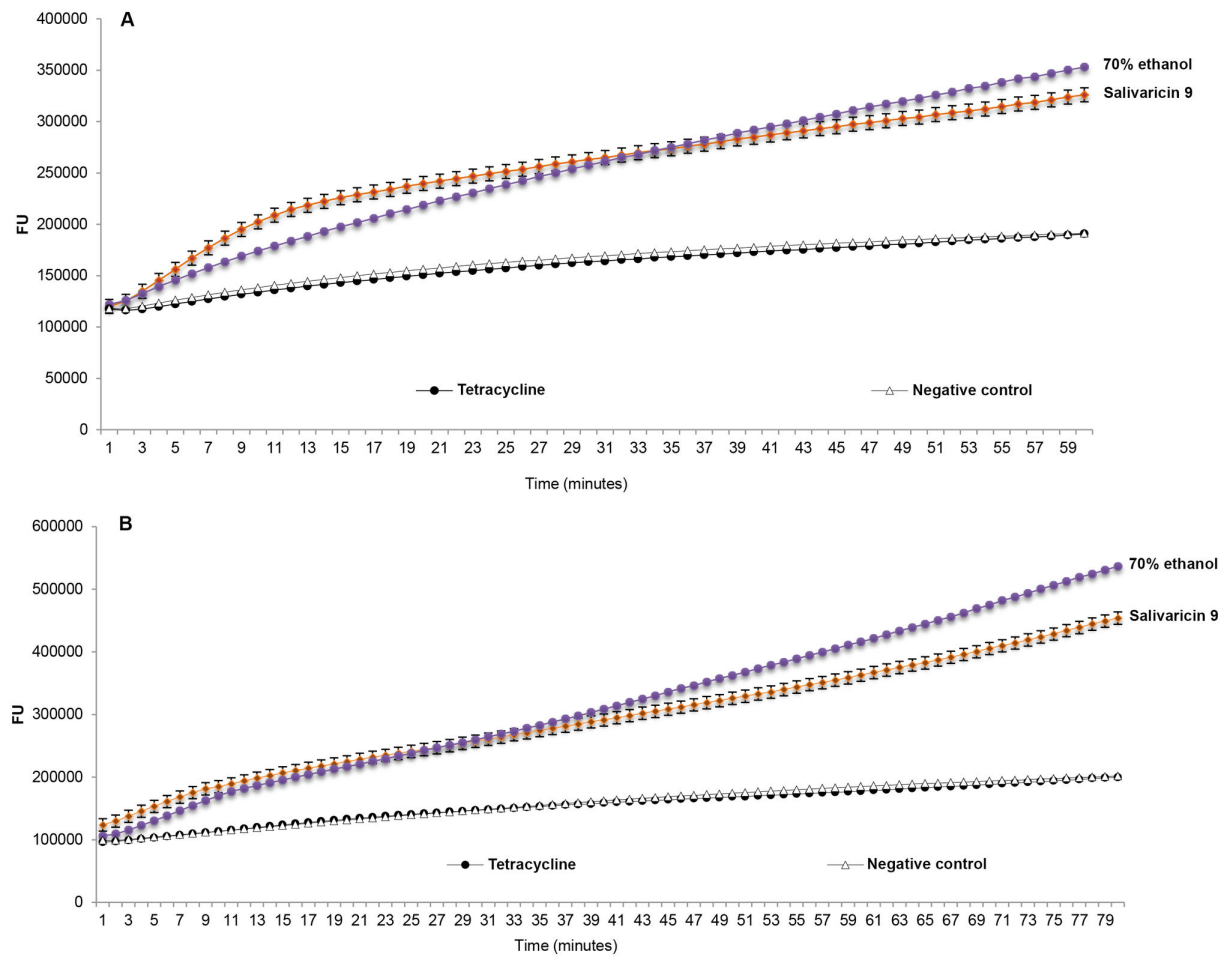
Membrane permeabilization assay of salivaricin 9. A: Salivaricin 9 permeabilization activity towards cytoplasmic membrane of *S. equisimilis*. B: Salivaricin 9 permeabilization activity towards cytoplasmic membrane of *Corynebacterium* *spp*. Negative controls comprise targeted bacteria without adding salivaricin 9. Positive control used 70% ethanol. Tetracycline did not show any permeability activity in this test.

The pure bioactive peptide was tested for inhibitory action against *Corynebacterium* *spp* and *Streptococcus equisimilis* ATCC 12388. The ability of salivaricin 9 to penetrate the cytoplasmic membrane of the *Corynebacterium* *spp* was greater compared to the permeabilization activity against *S. equisimilis*. The detected signal at 520 nm was the result of binding between the inner nucleic acid and SYTOX^®^ Green dye. Unlike SYTO9^®^ stain which can permeate both live and dead cells, the SYTOX^®^ Green stain is a high-affinity nucleic acid stain that easily penetrates cells with compromised plasma membranes and yet will not cross the intact membranes of live cells [29]. In this study the fluorescence signal from membrane-compromised bacteria labelled with SYTOX^®^ Green stain was detected by Real-Time PCR thermocycler since the fluorescence spectra of SYTOX^®^ Green (504/523) are close to those of SYBR green (494/521). Immediately after adding salivaricin 9 to the indicator strains the fluorescence started to increase gradually, indicating membrane permeabilization activity. In this assay 70% ethanol which is known to attack bacterial cell membranes [30]was used as a positive control. When tetracycline was assessed in this assay no significant fluorescence was detected due to its different mechanism of action. Tetracycline binds reversibly to the 30S ribosomal subunit to block the binding of the aminoacyl-tRNA to the acceptor site on the mRNA-ribosome complex and as a result protein synthesis is inhibited leading to bacteriostatic activity [31,32]. Figure 8 shows the membrane permeabilizing activity of salivaricin 9.

### Flow Cytometry analysis of membrane disruption

**Figure 9:**
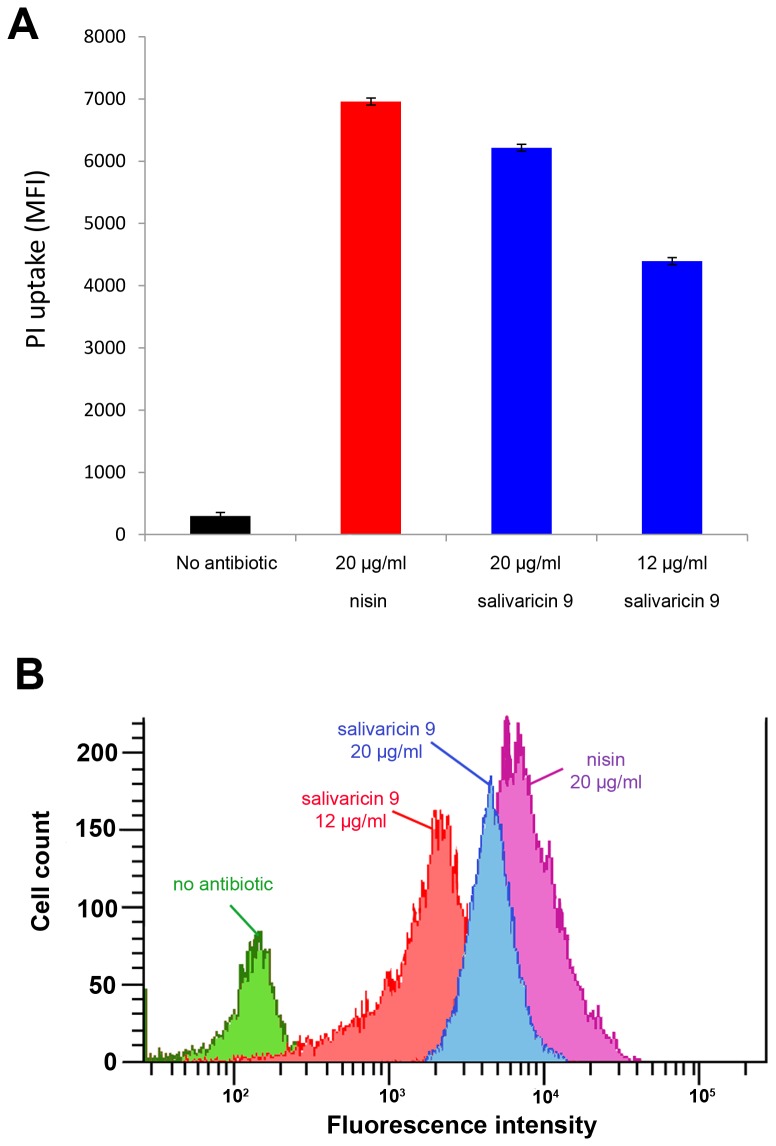
Flow cytometry analysis of pore-forming activity of salivaricin 9. Like nisin, salivaricin 9 alters the membrane permeability of *Micrococcus luteus* ATCC10240 as measured by propidium iodide (PI) uptake. (A) Average MFI of triplicate measurements for nisin at a concentration of 20µg/ml and a range of salivaricin 9 concentrations of 3-fold and 5-fold above its MIC value. (B) Representative histogram of cell count *versus* PI fluorescence intensity at antibiotic concentrations shown in panel A.

In this study potential membrane disruption in *Micrococcus luteus* ATCC10240 by salivaricin 9 and nisin was probed using the fluorescent dye propidium iodide (PI) which can enter dead cells in the presence of the pore forming agent, but cannot penetrate live cells with intact membranes. As expected, nisin (a pore forming lantibiotic) produced a large increase in cell-associated geometric mean fluorescence intensity (MFI). However, when cells were treated with salivaricin 9, an increase in fluorescence intensity occurred consistent with pore formation and loss of membrane integrity (Figure 9).

### Scanning Electron Microscopy

**Figure 10:**
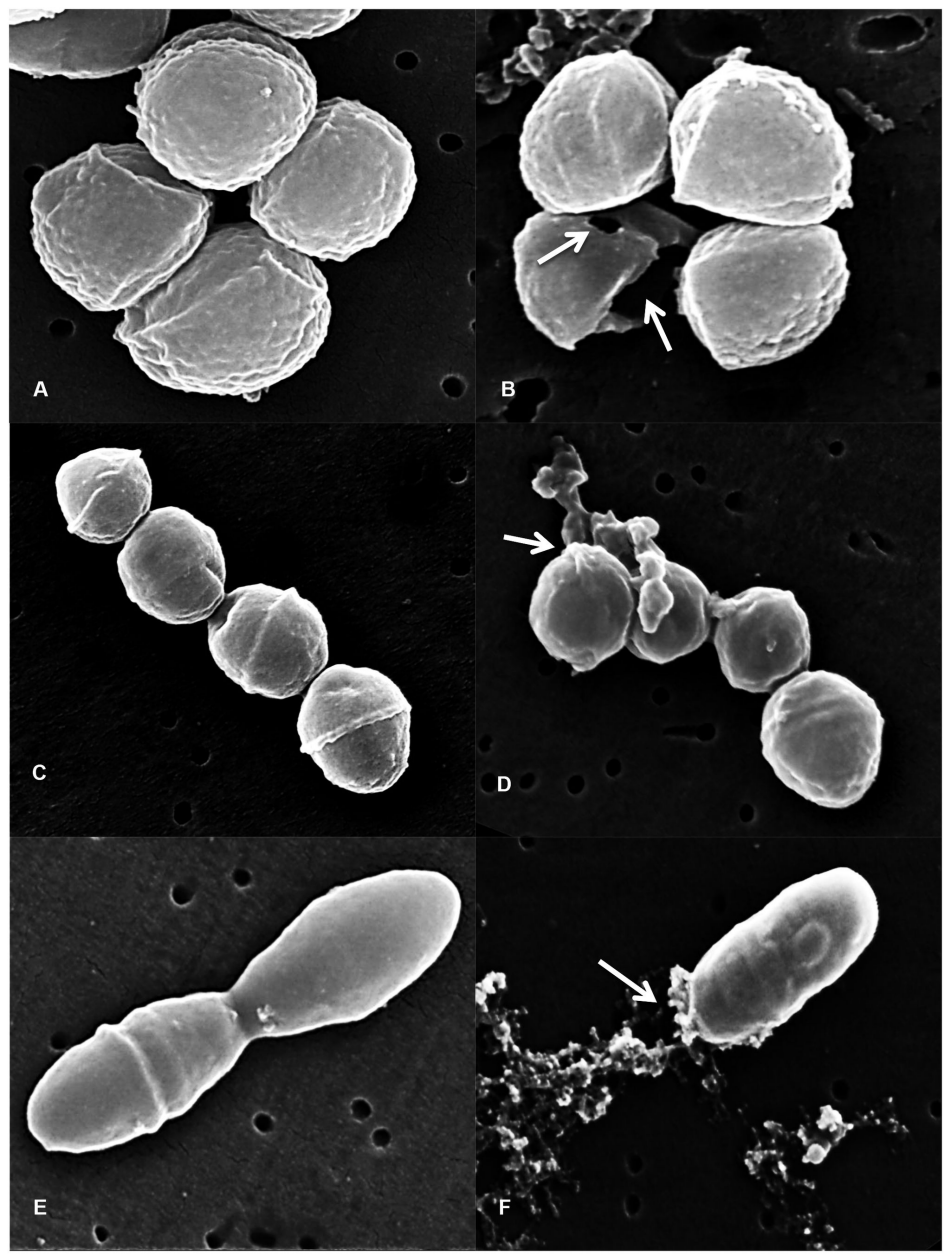
morphological changes of sensitive bacterial cells incubated with salivaricin 9. A: Untreated *Micrococcus luteus* used as a control. B: Morphological changes of *Micrococcus luteus* treated with salivaricin 9. C: Untreated *S. equisimilis* used as a control. D: Morphological changes of *S. equisimilis* treated with salivaricin 9. E: Untreated *Corynebacterium* *spp* used as a control. F: Morphological changes of *Corynebacterium* *spp* treated with salivaricin 9. White arrows indicate pores formed by salivaricin 9.

Salivaricin 9 caused major morphological changes in treated strains of Gram-positive bacteria. The pores formed after incubation with salivaricin 9 showed differences for the three tested strains. After a few minutes exposure to salivaricin 9, pores developed in the cell envelopes of *Micrococcus luteus* and *Corynebacterium* *spp.* indicating significant changes in their cell morphology. On the other hand *S. equisimilis* displayed less morphological damage following exposure to salivaricin 9. Nevertheless some pore formation was detected as the inner materials of the cells oozed through the pores resulting in cell death (Figure 10). 

### Salivaricin 9 stability

Salivaricin 9 retained biological stability when exposed to high temperature (90-100°C) for 30 minutes. The antimicrobial activity of salivaricin 9 was also retained after exposure to a wide pH range of 2 to 10. However salivaricin 9 appeared to be more stable in acidic and neutral conditions. Salivaricin 9 lost all antimicrobial activity when treated with proteinase K and peptidase (Table 4).

**Table 4:** Salivaricin 9 stability (thermal / pH/ proteinase K/ chemicals).

**Stability**	**Concentration**	**Inhibition zone**
Temperature (°C)		
4, 20, 30, 37, 40, 50, 60, 70, 80 for 1 hour		++++
90, 100 for 30 min		+++
121 for 20 min		-
pH value		
2- 7		++++
8-10		+++
11-12		-
Enzymes	1 mg.ml^-1^	
Proteinase		-
Proteinase K		-
Peptidase		-
Lyticase		++++
Catalase		++++
Detergents / Chemicals	1% (w/v)	
Tween 80		++++
Tween 20		++++
Tritone X100		++
SDS		++++
EDTA		++++
Urea		++++
NaCl		++++

(++++) : inhibition zone >20 mm; (+++) : inhibition zone =20 mm; (++): inhibition zone < 20 mm; (-): no bacterial inhibition. *Micrococcus luteus* was used as indicator. Salivaricin 9 titre: 800 AU/ml.

## Discussion

It has been reported that some bacteriocins including salivaricin A, salivaricin B [9,13] and mutacin [8] are controlled by quorum sensing mechanisms and they are better expressed when the producer bacteria grow profusely on solid media. This production strategy results in greater bacteriocin expression in solid culture media compared to that obtained from low density bacterial growth in liquid medium [33,34]. Usually, to produce these cell density-dependent bacteriocins, a freeze thaw method is used following growth of the inhibitory bacteria on solid or semi-solid media containing agar or agarose. In this study, a new method was developed to produce this type of bacteriocin in liquid medium. When bacteriocin production was first evaluated in liquid media using *S. salivarius* NU10 as the producer, no inhibitory activity could be detected. Most lantibiotic biosynthesis can be auto regulated by a signal transduction system for example bovicin HJ50 production by *Streptococcus bovis* [35], salivaricin A production by *S. salivarius* 20P3 [36] and nisin production by *Lactococcus lactis* [37]. To investigate at what stage of the bacterial growth the optimum quantity of salivaricin 9 can be recovered, a liquid medium system was used to enable estimates to be made of both the cell count and inhibitor titre. The induction procedure showed that salivaricin 9 production by strain NU10 is auto-regulated. When the culture was grown in the presence of supplementary salivaricin 9 (added at inducing levels) the yield of salivaricin 9 was increased to 1200 AU/ml (Figure 3). Feeding the induced culture with fresh medium helped to scale up the productivity while the culture itself worked as inoculum and inducer at the same time. Strains NU10 and YU10 are both PCR-positive for the salivaricin A structural gene, another auto-inducible lantibiotic shown to be produced by certain *S. salivarius* strains such as the prototype probiotic strain K12 (personal communication John Tagg). Crude peptide preparations from strains K12 and YU10 induced bacteriocin production in strain NU10. Strain K12 does not harbour *sivA* (the gene encoding salivaricin 9 production) and the induction activity of strain K12 toward NU10 is due to the production of salivaricin A by both strains rather than salivaricin 9 production. However, pure salivaricin 9 induced the inhibitor production in strain NU10 and YU10 (harbouring *sivA*) but not in strain K12 (*sivA*-negative). The use of cation exchange chromatography to purify lantibiotics has been described previously [38,39]. Salivaricin B has been purified using XAD-2 chromatography and RPHPLC [9] and also to separate the lantibiotics salivaricin A2 and B produced by *S. salivarius* *strain* K12. The current study is the first report of salivaricin 9 purification using SP Sepharose, a method resulting in improved recovery of salivaricin 9. Use of an XAD-16 resin was preferred over XAD-2 due to its higher capacity to bind salivaricin 9. XAD-2 particles had been used previously to adsorb salivaricin A [13] while amberlite XAD-4 was used to adsorb nisin [40]. The use of XAD-16 hydrophobic resin was a critical step in the protocol to achieve clear and desalted crude peptide. XAD-16 amberlite was used previously to adsorb LtnA1 and LtnA2 that comprise the two components of lacticin 3147 [41]. The FPLC system used in this study separated salivaricin 9 from the crude fraction prepared by XAD-16 chromatography. This purification protocol resulted in a single protein intensive band when Tris-Tricine SDS-PAGE was applied. Most of the impurities failed to bind to the SP sepharose resin and several peaks were detected after elution with a linear gradient of increasing salt concentration. The bioactivity test of all fractions showed that three active fractions corresponding to a single peak were eluted at a NaCl concentration of 23%. There is a lack of information about the mechanism of action of lantibiotics produced by oral streptococci. The permeabilization activity of some lantibiotics toward gram positive bacteria has been studied previously [26]. The depolarization of the cytoplasmic membrane of gram positive bacteria by salivaricin 9 was assessed by the de-quenching of SYTOX^®^ Green fluorescence in this study. Flow cytometry analysis was used to confirm the permeability activity of salivaricin 9 as a pore former. Both molecular probes SYTOX^®^ Green and propidium iodide are impermeable stains and yet cannot penetrate cells with intact membranes [42]. Propodium iodide has been used previously to check for pore formation by some bacteriocins, antibiotics and other antimicrobial agents [43-46]. Treating *Micrococcus luteus* ATCC10240 cells with salivaricin 9 allowed the (PI) dye to enter the cells through damaged cytoplasmic membranes. However, there is correlation between MFI and lantibiotic concentration. The MIC value of salivaricin 9 for this strain (4µg/ml) did not show significant increase in the MFI after 30 minutes of incubation. However, increases of 3-fold (12µg/ml) and 5-fold (20µg/ml) above the MIC value showed significant MFI increments. An overview of this data suggests that salivaricin 9 is able to form pores in bacterial membranes which will lead to loss in membrane integrity of the cells. This conclusion may explain the morphological changes that salivaricin 9 can cause in the targeted cells. Type A lantibiotics (e.g. nisin and epidermin) are amphipathic and elongated molecules which mainly act by forming pores into the cytoplasmic membrane of the targeted bacterial cell [25,47,48]. Binding to lipid II is an initial factor inducing a transmembrane orientation of the pore forming lantibiotic [25]. At some stage lantibiotics such as nisin can form pores and can inhibit cell wall biosynthesis when they bind to the peptidoglycan precursor lipid II [49]. Salivaricin 9 showed similar characteristics in this study to other lantibiotics in bringing about membrane permeabilization by pore formation in the cytoplasmic membrane. Further work is now in progress to evaluate the mechanism of binding of salivaricin 9 to lipid II precursor by using fluorimetric spectroscopy. Mass production using cloning and expression techniques is also being investigated so that the lantibiotic can be commercialized as a biopharmaceutical.

## Conclusions

Salivaricin 9 produced by strain *S. salivarius* NU10 isolated from a Malaysian subject was produced in liquid medium using an induction method. XAD-16 and cation exchange chromatography were used to purify the active agent. MALDI-TOF analysis showed that the pure peptide has a molecular weight of 2560 Da. Salivaricin 9 displayed bactericidal and bacteriolytic activity against and induced cytoplasmic membrane permeability in susceptible bacterial cells. Pore formation activity was detected in sensitive strains incubated with salivaricin 9. This study has revealed additional information on an antimicrobial peptide produced by *S. salivarius* that can expedite the further development of specifically targeted novel antibiotics in this era of increasing antibiotic resistance. 

## Materials and Methods

### Bacterial strains and media

*S. salivarius* NU10 and YU10 were isolated from two healthy Malaysian subjects and then deposited in NCBI Gene Bank under accession numbers KC796011 and KC796012 respectively. Strains NU10 and YU10 were isolated from the two subjects who were required to sign a consent form to isolate *S. salivarius* from the tongue surface using sterile cotton swabs. No approval for sampling from tongue surface is required from the IRB as a pro forma for written consent from the subject is approved by the IRB. The ethics committee IRB Reference No. is DF OP1304/0019 (P) for our Institution (University of Malaya). We have discussed this with the ethics committee (IRB) and the protocol complied with Good Laboratory Practices and no IRB approval is required prior to sampling in this cases. This work was entirely done in our own home country Malaysia within the University campus that includes a university hospital. *S. salivarius* K12 a commercial probiotic (BLIS Technologies Ltd, New Zealand) was provided by John Tagg (University of Otago, New Zealand) for use as a positive control. Indicator strains used in this study are listed in Table 3. All the indicator strains are either purchased from American Type Culture Centre (ATCC) or were taken from the culture collection of Microbial Biotechnology Laboratory, Division of Microbiology, Institute of Biological Science, Faculty of Science, University of Malaya, Kuala Lumpur, Malaysia. Todd Hewitt broth (THB) was used to propagate all the bacterial strains in this study. Mitis Salivarius agar (MSA) was used to isolate pure colonies of *S. salivarius* strains. M17 broth supplemented with 2% yeast extract, 1% sucrose, 0.1% calcium carbonate (M17YESUCa) was used to produce salivaricin 9 in a liquid medium system. Columbia agar base supplemented with 5% whole human blood and 0.1% calcium carbonate (BACa) was used in simultaneous antagonism tests. All media was purchased from Difco, Becton Dickinson, Sparks, Md., USA.

### Simultaneous antagonism assay

To check for antimicrobial activity, *S.salivarius* NU10 was stabbed into blood agar plate supplemented with 0.1% calcium carbonate previously seeded with a lawn of *Micrococcus luteus*. *S. salivarius* K12 (producer of salivaricin A2 and salivaricin B) was used as a positive control (Figure 1).

### DNA isolation and detection of *sivA* structural gene encoding salivaricin 9 production

*S. salivarius* strain NU10 was grown aerobically in THB for 18 hours at 37°C. Cells were collected by centrifugation at 8000 x g for 3 minutes and washed twice with 0.85% NaCl before suspending in lysis buffer comprising of 20 mM Tris-HCl at pH 8.0, 2 mM sodium EDTA, 1.5 % Triton X-100 and 50 mg/ml lysozyme. DNA was isolated and purified using DNeasy^®^ Blood & Tissue kit (QIAGEN) following the manufacturer’s instructions. To detect *sivA*, primers sivF (5`-AAAAAGGCGCTTCTATATCCATGA-3`) and sivR (5`-ATCTTTACCTCAAACTTTTAAGTCCATT-3`) were used as described previously [12]. Primer pair SalAUS (5`-GTAGAAAATATTTACTACATACT-3`) and SalADS (5`-GTTAAAGTATTCGTAAAACTGATG-3`) were used to detect *salA* encoding salivaricin A production in strains NU10 and YU10 as described previously [11]. *sivA* of strain NU10 was sequenced and assembled using DNA baser V.3 software and analysed using *in silico* DNA to protein translation tool [28] (Figure 2). 

### Salivaricin 9 induction assay

The bacteriocin-inducing activities present in crude extracts of *S. salivarius* strains NU10, YU10, K12 and in FPLC-purified salivaricin 9 peptide fractions having anti-*Micrococcus luteus* activity was investigated in this study. The crude extracts of producer cells designated as BLIS-NU10, BLIS-YU10 and BLIS-K12 were isolated by acidic methanol extraction of the producer cells as described previously [11,12]. These crude BLIS extracts had the potential to contain at least two lantibiotics on the basis of PCR assessment for their content of lantibiotic structural genes. Strains NU10 and YU10 had the structural genes for salivaricin 9 and salivaricin A and strain K12 had salivaricin A and salivaricin B structural genes. Salivaricin 9 was shown to be auto-regulated whereby a small amount of the active peptide can induce enhanced production in a large scale production system. Colonies from an 18 h culture of each *S. salivarius* strain NU10, YU10 and K12 grown anaerobically on BACa agar was used to inoculate 10 ml of M17YESUCa broth and incubated again under the same conditions. The cells were then collected by centrifugation at 18000 x g for 5 min and washed three times in saline buffer to reduce the levels of cell surface-associated lantibiotic before suspending in 10 ml of the same buffer. 1.5 ml Eppendorf tubes containing 0.9 ml of M17YESUCa were inoculated with 0.1 ml of the washed cells before 0.05 ml of the test sample (BLIS-extracts or pure salivaricin 9 of titre 4 AU/ml) was added and these tubes marked as “induced”. Tubes with no BLIS-extract or salivaricin 9 added were marked as “control” (uninduced). Both induced and control tubes were incubated anaerobically for 18 h before 0.05 ml of the test sample (BLIS-extract or salivaricin 9 of titre 4 AU/ml) was added to the control tube. 50µl aliquots of each sample (induced and control) were used to test for antimicrobial activity using the well diffusion assay. Induction of salivaricin 9 or salivaricin A production was recorded as inhibitory zones surrounding samples from the induced tubes but not the control tubes. Nisin producing *Lactococcus lactis* strain ATCC11454 was also tested for bacteriocin-inducing activity as a positive control (Table 1).

### Production of salivaricin 9 in liquid medium

Usually bacteriocins produced by *S. salivarius* can be isolated from cultures grown on solid medium using the freeze thaw method described previously [9]. Strain NU10 is also able to produce the bioactive peptide in solid phase medium and showed extremely weak expression of inhibitory activity in liquid media. To overcome this limitation, a new technique was used to enhance the production of salivaricin 9 lantibiotic in liquid media. Strain NU10 was grown on MSA for 18 h under anaerobic condition at 37°C before one colony (dome-shape in appearance) was used to inoculate 20 ml of M17YESUCa broth and incubated under the same conditions mentioned above on an orbital shaker at 150 rpm. The cells were collected by centrifugation at 6000 x g for 5 min at 4°C and then washed twice in 0.85% NaCl prior to dissolving in 20 ml of the same solution. The cell suspension was used to inoculate 50 ml of fresh M17YESUCa broth at 37°C in anaerobic condition for 20 h. The resulting culture was fed with fresh 50 ml of the same medium and 0.05 g/ml of pure salivaricin 9 was added as an inducer before the 100 ml culture was incubated for another 18 h. 80 ml of the final culture was used to inoculate 820 ml of M17YESUCa broth. The pH was adjusted to 6.5 using concentrated HCl. CFU and AU values during production were recorded (Figure 3).

### Salivaricin 9 purification and determination of minimal inhibitory concentration (MIC)

An induced culture of *S. salivarius* NU10 was centrifuged at 8000 x g to pellet the cells. The cell free supernatant (CFS) was filtered through 0.2 µm sterilized cellulose membrane (Millipore) to ensure that all cell debris was removed. The filtered crude bacteriocin preparation was passed through 100 ml XAD-16 particles (Sigma) packed in a glass column. The column was washed with 400 ml of distilled water followed by 200 ml of 70% methanol before the active fraction was eluted using 200 ml of 95% methanol (adjusted to pH 2) at a flow rate of 15 ml/min. The methanol was removed using a rotary evaporator and the resulting peptide preparation was lyophilized and stored at -20°C. The lyophilized peptide pellets were dissolved in 20 mM sodium phosphate pH 5.8 (binding buffer). Then the sample was injected into a fast protein liquid chromatography (FPLC) system (ÄKTA Purifier™) using HiTrap SP FF 5ml strong cation exchanger column (GE Healthcare) equilibrated with the same buffer at a flow rate of 1 ml/min. Then the column was washed with 10X column volume of the binding buffer before salivaricin 9 was eluted using a linear gradient of 0 to 1M NaCl in 20 mM sodium phosphate buffer at pH 5.8 (Figure 4). All fractions were tested for inhibitory activity using *Micrococcus luteus* as the indicator. Purification steps, fold and yield are listed in Table 2. MIC values of pure salivaricin 9 against a set of bacterial indicator strains were determined by following the National Committee for Clinical Laboratory Standards (NCCLS) (Table 3).

### Tris-Tricine SDS page

The active fractions obtained by FPLC were subjected to 16.5% sodium dodecyl sulphate (SDS) electrophoresis as described previously [50] using a Tris-Tricine buffer system. Mini-Protean Tetra Cell (Bio Rad) was used according to manufacturer’s instructions. Dual Xtra protein marker (Bio Rad) was used to estimate the molecular weight of the pure salivaricin 9. The gel run was at 125 V for 45 min and stained using SimplyBlue™ SafeStain (Life Technologies-Invitrogen). After de-staining, the gel was visualized and the molecular weight estimated (Figure 5).

### Identification of the antimicrobial peptide

The FPLC purified peptide was subjected to matrix-assisted laser desorption-time of flight (MALDI-TOF) mass spectrometry (MS) at the Medical Biotechnology Laboratory, Faculty of Medicine, University of Malaya.

### Membrane permeabilization assay

The membrane permeabilization activity of salivaricin 9 towards the cytoplasmic membrane of targeted bacterial cells was examined using SYTOX^®^ Green dye. A log phase culture of the targeted bacteria was centrifuged at 9000 x g for 5 minutes and then the cells were washed 3 times with 10 mM HEPES buffer before re-suspending in the same buffer. The CFU were counted to be 9 x 10^5^. 5µM of SYTOX^®^ Green (Invitrogen™) was added to the bacterial suspension after which 100µl of this mixture was transferred to a 96 well PCR plate before 100µg/ml of salivaricin 9 was added to the mixture (SYTOX^®^ Green + bacterial cells). Wells containing only bacterial cells and SYTOX^®^ Green were designated as negative control wells. 70% ethanol was used as the positive control. Tetracycline (1.5µg/ml) was also tested in this assay as a known antimicrobial having a different mode of action. The fluorescence generated from the binding between SYTOX^®^ Green and nucleic acid of the dead bacterial cells with compromised membranes was detected at 520 nm and 37°C exposing for over a period of 60 and 80 minutes for *S. equisimilis* and *Corynebacterium* *spp* respectively (Figure 8). The experiment was performed in triplicate and raw data was analyzed using Microsoft Excel software. 

### Flow Cytometry analysis of membrane disruption

For membrane integrity assay using the dye propidium iodide (PI), cultures of *Micrococcus luteus* ATCC10240 were grown over night at 37°C in THB supplemented with 0.5% glucose (THBG) and then diluted with fresh THBG to an OD_600_ of 0.1. The cells were centrifuged at 4000 x g for 5 minutes at 4°C and washed twice with washing buffer (2 mM HEPES + 2 mM glucose) before re-suspending in the same buffer and adjusting to OD_600_ 0.1. The cells were exposed to salivaricin 9 (12, 20 µg/ml) or nisin (20 µg/ml) (Sigma) and PI (final concentration 25 µM) before incubating for 30 minutes at 37°C. Nuclease-Free Water (Promega, USA) was added instead of antibiotic and served as a negative control. Samples were analyzed using BD FACSCanto^TM^ II flow cytometer using excitation at 488 nm and with the standard filter setup. Each sample was examined in triplicate and the geometric mean fluorescence intensity (MFI) of gated cell populations was calculated (Figure 9).

### Scanning electron microscopy (SEM)

To study the morphological changes of the targeted bacterial cells after treating with pure salivaricin 9, indicator microorganisms were grown in THB to reach the exponential phase of growth at (OD_600_ 0.7) and then the bacterial cultures were diluted to OD_600_ 0.2 before pure salivaricin 9 was added to the cultures and incubated at 37°C for 260 minutes. After incubation, the bacterial pellets were centrifuged at 6000 x g for 10 minutes at 4°C and washed twice with phosphate buffer saline PBS at pH 7 before the samples were fixed with 8% glutaraldehyde in 1:1 (v/v) Sorensen’s phosphate buffer for one hour. 1:1 (v/v) of Sorensen’s phosphate buffer and water mixture was applied before the samples were fixed with 4% OsO_4_ mixed with 1:1 (v/v) H_2_O. After overnight incubation, samples were washed with deionized water for 15 minutes and then dehydrated in increasing concentrations of ethanol. Dehydration with ethanol-acetone mixture followed by pure acetone was applied before critical point drying (CPD). After sample preparation JEOL JSM-7001F Scanning Electron Microscopy was used to visualize the morphological changes of bacterial cells treated with salivaricin 9 compared with non-treated cells (Figure 10).

### Stability tests on salivaricin 9

Heat stability was studied by exposing pure salivaricin 9 to different temperatures for one hour after which the sample was centrifuged and the supernatant tested for inhibitory activity. The stability of the peptide at different pH values was also evaluated and tested for inhibitory activity. Specific enzymatic effects of denaturation on salivaricin 9 were tested using two wells of 6mm diameter each separated by a distance of 4 mm in CAB plates. One well was filled with 50µl of pure salivaricin 9 while the other was filled with 50µl of the tested enzyme. The plate was incubated at 50°C for 2 hours and then incubated at 37°C overnight before seeding the indicator strain onto the agar surface using a cotton swab. Then the plate was re-incubated at 37°C for 18 hours and the zones of inhibitions that appeared indicated residual activity of salivaricin 9 while absence of inhibition zones indicated the denaturation of the antimicrobial peptide by the applied enzymes namely proteinase K, peptidase, lyticase and catalase. The stability of salivaricin 9 to treatment with the chemicals EDTA, SDS, urea, NaCl and β-mercaptoethanol was examined by adding 1% of these chemicals to the bacteriocin followed by incubation for two hours at RT before the samples were centrifuged and the supernatant tested as mentioned above. If the chemical was a solvent such as β-mercaptoethanol, then the solvent was evaporated before testing for antimicrobial activity (Table 4).
